# Ascending colon cancer metastasized to the right testicle: a case report

**DOI:** 10.1186/s13256-024-04587-z

**Published:** 2024-06-27

**Authors:** Qingqiang Gao, Yuanzhi Li, Leilei Zhu

**Affiliations:** 1 Department of Andrology, Nanjing Drum Tower Hospital, The Affiliated Hospital of Nanjing University Medical School, Nanjing, China; 2 Department of Intensive Care Unit, Nanjing Drum Tower Hospital, The Affiliated Hospital of Nanjing University Medical School, Zhongshan Road No. 321, Nanjing, Jiangsu, 210008 China; 3 Department of Urology, Wuxi People’s Hospital Affiliated to Nanjing Medical University, Qingyang Road No. 299, 214000 Wuxi, Jiangsu People’s Republic of China

**Keywords:** Ascending colon carcinoma, Case report, Testicular metastasis, Hemicolectomy, Radical orchiectomy

## Abstract

**Background:**

Testicular metastasis from malignant solid tumors is extremely rare. It is usually found by chance during autopsy or pathological examination of testicular specimens. Therefore, we consider it necessary to report our patient’s case of testicular metastasis from colon cancer.

**Case presentation:**

We report a 61-year-old Han Chinese male patient who presented to our clinic with progressive painless swelling of the right testicle for 2 years. Positron emission tomography–computed tomography scans showed increased 18F-fluorodeoxyglucose metabolism in the right testicle, possibly owing to distant metastasis. His previous medical history suggested that he had undergone laparoscopic-assisted right hemicolectomy for ascending colon cancer 4 years ago. Considering the ascending colon cancer metastasis to the right testicle, we performed a right radical testicular resection through an inguinal approach. Postoperative histological examination showed intestinal metastatic adenocarcinoma.

**Conclusion:**

Colon cancer metastasis to the testes is uncommon. The clinical and imaging manifestations of this tumor are nonspecific, so the diagnosis relies on postoperative pathology. If testicular metastasis is found, treatment principles for advanced colon cancer should be followed.

## Background

Testicular cancer is the most common cancer in young men, presenting as a single testicular mass, with a high cure rate through radical orchiectomy [1]. However, previously reported cases have shown that testicular mass is the first clinical manifestation of underlying malignancy [2]. Testicular metastasis from malignant solid tumors is extremely rare, with reported incidences of testicular metastasis in autopsy or surgical specimens ranging from 0.02% to 2.5% in patients with different malignant tumors [3]. In addition, the nonspecific characteristics of the primary tumor of testicular metastases pose a challenge for diagnosis. According to previously reported cases of testicular metastases, the tumors usually occur in men with prostate cancer [4], malignant melanoma [5], renal cell carcinoma [6], colonic carcinoma [7], lung carcinoma [8], etc. However, the specific mechanisms of testicular metastasis in these tumors are not fully understood. There are several hypotheses regarding testicular metastatic carcinoma. These hypotheses include lymphovascular invasion, transperitoneal implantation, and direct spread along the vas deferens.

In this case report, we present a 61-year-old man with ascending colon cancer that metastasized to the right testicle. The patient underwent right hemicolectomy 4 years ago. The patient consented to the collection and use of his data for research purposes.

## Case presentation

**Fig. 1:**
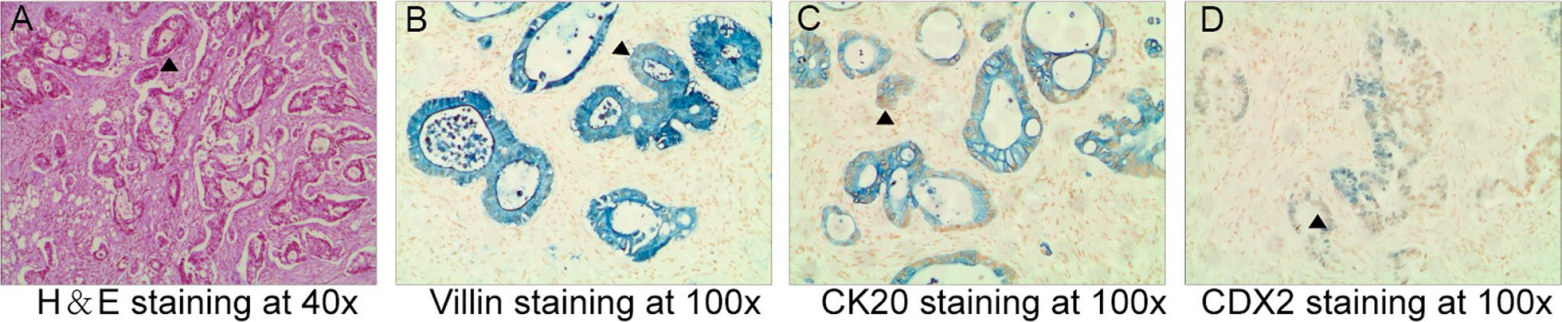
Histological examination of the orchiectomy specimen. **A** Hematoxylin and eosin staining showed metastatic adenocarcinoma within the testis. **B**–**D**. Immunohistochemically the tumor cells were positive for Villin, CK 20, and CDX2, respectively (arrows)

A 61-year-old Han Chinese male patient was admitted to our hospital owing to progressive painless swelling of the right testicle for 2 years. Upon admission, we conducted a comprehensive investigation into his medical history. According to the records, he underwent laparoscopic-assisted right hemicolectomy for ascending colon cancer at another hospital 4 years ago and received postoperative chemotherapy. However, owing to the loss of previous case records, the specific treatment plan remains unknown. He has no medical history of hypertension, type 2 diabetes, hyperlipidemia, coronary heart disease, trauma, or any other relevant conditions. The patient does not have symptoms of polyuria, urgency, dysuria, or hematuria. Additionally, he has no history of tobacco or alcohol use and no family history of malignancies. After taking the patient’s medical history, we conducted a physical examination, including measuring blood pressure, weight, and height. The patient’s physical examination revealed stable vital signs with normal heart and lung sounds (temperature 37.0 °C, heart rate 75 beats/minute, blood pressure 118/70 mmHg, respirations 18 times/minute). His abdomen was soft without tenderness or organ enlargement observed. The neurological assessment of the patient revealed no irregularities. Genital palpation identified a solid lump in the right hemiscrotum along with an enlarged testis measuring approximately 10.0 × 4.0 × 3.0 cm. His laboratory results were normal, including blood tests, urine tests, liver function tests, kidney function tests, total testosterone, alpha-fetoprotein, carcinoembryonic antigen, and CA125. An electrocardiogram showed no abnormalities. Pelvic magnetic resonance imaging (MRI) in another hospital suggested that after colon cancer surgery, local denseness with uneven enhancement was found in the pelvic region between the rectum and the prostate, seminal vesicle area, and adjacent pelvic wall, suggesting metastasis, and abnormal signal in the right testicle. Positron emission tomography–computed tomography (PET/CT) scanning showed abnormal glucose metabolism in the abdominal cavity, increased lymph nodes, and unclear decomposition of the lymph nodes between the rectum and the prostate, seminal vesicle area, and adjacent pelvic wall, suggesting metastasis. The right testicle showed high 18F-fluorodeoxyglucose metabolism, which may be from distant metastasis. The patient did not undergo any medical intervention prior to his diagnosis. In this case, we performed radical right testicular resection through the inguinal approach. The resected testicular specimen showed a solitary gray-white testicular mass. Histological examination showed metastatic adenocarcinoma from the intestine, without spermatic cord or epididymal invasion (Fig. 1).

Taking into account these results, systemic therapy was initiated approximately 2 weeks after surgery, including capecitabine and irinotecan (1.5 g of capecitabine taken orally twice a day and 0.3 g irinotecan intravenously). He underwent six cycles of chemotherapy. Liver metastasis was found at the patient’s follow-up visit 6 months after surgery. However, a telephone follow-up showed that the patient survived for more than 18 months after surgery.

## Discussion

The metastasis of colorectal cancer to the testis is rare. In this report, we present a case history, diagnostic procedures, and treatment for this occurrence. On the basis of our findings, we recommend routine testicular examination for colorectal cancer patients to reduce metastatic disease incidence.

Testicular cancer is the most common cancer in young men. It usually occurs in the early stage (clinical stage I) and is highly curable through radical orchiectomy. The 5-year survival rate for all stages of testicular cancer is 96.6% [1]. However, the testes may also be the site of metastatic disease. Cases of metastasis to the testes are rare and are usually discovered by chance during autopsy or pathological examination of the testicular specimen. Some cases of metastasis from colon cancer to the testes have been reported previously [7, 9]. Colon cancer metastasis to the testes is uncommon. And when metastasis to the testes is the first manifestation of colon cancer, it is even rarer [10]. Previous reviews have shown that the median age of colon cancer metastasis to the testes is 52 years old, ranging from 18 to 87 years old, and the average survival time from diagnosis to survival in patients with colon cancer metastasis to the testes is 6–12 months [11]. The metastatic tumor usually presents as a solitary unilateral nodule and may be similar to the primary testicular tumor. It poses a diagnostic challenge in distinguishing between primary and secondary testicular cancers. However, some aspects of testicular metastasis are of clinical and pathological importance.

However, the specific mechanism of testicular metastasis from colorectal cancer is not fully understood. The various pathways of colorectal cancer metastasis to the testes have been thoroughly discussed [3]. These include: (1) retrograde lymphatic spread, (2) direct invasion or expansion, (3) arterial and venous embolization of the tumor, and (4) it is worth noting that different types of colorectal cancer may have different pathways to the testes. We speculate that the occurrence of testicular metastasis is related to a variety of factors, including: the location of colorectal cancer, surgical specimens with positive lymph nodes, peritoneal metastasis, intraoperative manipulation to carry tumor cells to the testes, the proliferation ability of tumor cells, and accompanying symptoms, such as penile space effusion.

The prognosis of metastatic colorectal cancer is poor. The 5-year survival rate for patients with early-stage colorectal cancer is approximately 90%, but the 5-year survival rate for patients with regionally spread tumors drops sharply to 65%, and the 5-year survival rate for patients with advanced disease and distant metastases drops to 10% [12]. The conventional treatment strategies for metastatic colorectal cancer include systemic chemotherapy, radiotherapy, and surgery. Usually, the goals of surgery for metastatic colorectal cancer are to relieve symptoms, control tumor growth, and improve survival. Resection of liver or lung metastases from primary colorectal cancer can improve survival in these patients [13]. We speculate that resection of testicular metastases in these patients has a similar therapeutic effect. Furthermore, there is no evidence that postoperative chemotherapy can improve survival in these patients with colorectal testicular metastases. Perhaps we are seeing testicular metastases in these colorectal cancer patients, and in fact, other distant metastases have occurred. Finally, we suggest that postoperative chemotherapy can prolong the survival time of these patients.

## Conclusion

The metastasis of colon cancer to the testes is rare. When evaluating testicular symptoms in colorectal cancer patients, it is important to consider the possibility of metastasis from the colorectal region. Clinical and imaging characteristics are nonspecific, requiring postoperative pathology and immunohistochemistry for a definitive diagnosis. Surgical goals include relieving symptoms, controlling tumor growth, and improving survival.

## Data Availability

All data used during the present study are available from the corresponding author upon reasonable request.
